# Morphological relationship between the superior cervical ganglion and cervical nerves in Japanese cadaver donors

**DOI:** 10.1002/brb3.619

**Published:** 2016-12-29

**Authors:** Kazuyuki Mitsuoka, Takeshi Kikutani, Iwao Sato

**Affiliations:** ^1^ Department of AnatomyThe Nippon Dental University School of Life Dentistry at TokyoTokyoJapan; ^2^ Division of Clinical Oral RehabilitationThe Nippon Dental University Graduate School of Life DentistryTokyoJapan; ^3^ Department of Clinical Oral RehabilitationThe Nippon Dental University School of Life Dentistry at TokyoTokyoJapan

**Keywords:** ganglion cell, glossopharyngeal nerve, shrunken neuron, superior cervical ganglion, vagus nerve

## Abstract

**Introduction:**

There are various communications between the superior cervical ganglion (SCG) and the vagus and glossopharyngeal nerves. However, little information exists concerning the origin of these sympathetic ganglion branches at the superior, middle, and inferior regions of the human SCG. The aim of this study was to describe the human SCG in a morphometric manner with the communication with cranial and cervical nerves and supply.

**Methods:**

This study characterized 72 SCG samples from 54 elderly Japanese human cadavers (30 males, 24 females; 65–100 years old). The SCG size (length, width, and thickness) and location were measured from the jugular foramen. We also defined the communication branches of the SCG to the vagus, glossopharyngeal, cervical, and accessory nerves at three regions (superior, middle, and inferior regions) of the SCG. Finally, we examined the arrangement and origin of the branch communications in detail and confirmed our observations, using histological sections of the SCG.

**Results:**

The SCG in all cadaver donors was detected at the C2 and C3 vertebra levels. The number of SCG branches supplied the communicating branches, such as the carotid branch, communicating branch of the vagus nerve, and glossopharyngeal nerve, were frequently detected in the superior region of the SCG (χ^2^ = 587.72, df = 26, *p *<* *.001). The number of ganglion cells with a large number of neurons per unit area (1 mm^2^) was most often found in the middle region with shrunken neurons of the SCG compared with other regions.

**Conclusion:**

The communication branches of the SCG are mainly connected to the vagus and glossopharyngeal nerves. Characterizing these branches can provide useful data for head and neck ganglion block and surgical treatments.

## 1 Introduction

Previous investigators have indicated that the communication branches of the cervical ganglion are mainly found between the inferior cervical ganglion of the vagus nerve and superior cervical ganglion (SCG) (Braeucker, 1923; Fick, 1926; Siwe, l931; Hoffman, 1957). The SCG has some branches that communicate with the branch of the vagus nerve (the internal carotid nerve and external carotid arteries, superior laryngeal nerve, superior cervical cardiac branch, thyroid branch, and pharyngeal branch) and branches of the glossopharyngeal nerve (carotid branch, internal branch and external carotid artery, the nerve to the stylopharyngeus, and nerves to the pharyngeal plexus) (Robinson, 1922; Standring, 2005). Sato, Sato, and Shimada (1997) defined a rare complex communication between the vagus nerve and the SCG. Communication between the superior and inferior laryngeal nerves has been recorded in 3 of 19 (Nordland, l930) and in 10 of 12 cases (Lemere, 1932). There are various communications of the SCG with the vagus and glossopharyngeal nerves. However, little information exists concerning the origin of these branches at the superior, middle, and inferior regions of the SCG. The morphological properties also indicate the presence of efficient pharmacokinetic parameters to treat headache and facial pain compared with the stellate ganglion block (Fazliogullari, Kilic, Karabulut, & Yazar, 2016; Harris, Hamid, Rosenquist, & Schultz‐Stubner, 2006). The location of the SCG is an important needle position in anesthetic treatment (Elias, 2000; Siegenthaler, Haug, Eichenberger, Suter, & Moriggl, 2013; Wirz, Wartenberg, Nadstawek, & Kinsky, 2008). The application of local opioid analgesia at the SCG represents a suitable and simple treatment option for neuropathic facial pain (Elsner, Radbruch, Gaertner, Straub, & Sabatowski, 2006). The morphology of the SCG serves as a guide for nerve blockage information and as a useful surgical treatment for the head and neck. The SCG may supply many branches that arborize with branches of some cranial nerves; however, the formation of these communication branches of the SCG is unknown according to previous reports.

Therefore, we investigated the communication of SCG branches at the macroscopic level in detail. These observations provide useful data for clinical treatments in the head and neck.

## 2 Materials and methods

### 2.1 Macroscopic observations

We examined 72 sides of 54 adult Japanese cadaver donors (30 males and 24 females) to obtain details of the passage or supply of SCG branches and communications with cranial and cervical nerves in the neck. Thirty male cadavers that were 59–95 years old (mean 77.6 ± SD 10.3 years) provided 39 specimens; 22 specimens were from the right side, and 17 were from the left side. Twenty‐four female cadavers that were 61–96 years old (mean 82.4 ± 9.27 years) provided 33 specimens; 15 specimens were from the right side, and 18 were from the left side. Subjects with neck disease and partially damaged or already dissected SCG were excluded from our study. Using forceps, we removed the entire mucous membrane and connective tissues of the neck. We clearly identified the course of communication of branches of the SCG and vagus nerve, glossopharyngeal nerve, accessory nerve, and cervical nerve and internal carotid artery, internal jugular vein, external carotid artery, pharynx, and larynx. We measured six points: the maximum length of the SCG (L‐SCG), maximum width of the SCG (W‐SCG), maximum thickness of the SCG (T‐SCG), distance between the external opening of the jugular foramen and hyoid bone (JF‐HB), distance between the jugular foramen and tip of the SCG (JF‐SCG), and distance between the jugular foramen and carotid bifurcation (JF‐CB), using a vernier calipers. We also classified the branch type at the superior, middle, and inferior regions of the SCG in our examined specimens. We counted the number of SCG branches, except for very fine ramie branches (<0.1 mm), at three regions of the SCG. Superior, middle, and inferior regions are defined the length of three equal parts of SCG.

### 2.2 Histological observations

We examined the arrangement and origin of branch communication in detail and confirmed our observations, using sections of the SCG. Paraffin‐embedded blocks and sections of the SCG for histochemistry were obtained from Japanese cadaver donors. SCG samples were fixed with tissue fixative (Genostaff Co., Ltd. Tokyo, Japan), embedded in paraffin, using Genostaff's proprietary procedures and sectioned at approximately 5 μm. The tissue sections were deparaffinized with xylene and rehydrated through a series of ethanol solutions in PBS. The sections were stained with Mayer's hematoxylin and eosin (Muto, Tokyo, Japan), dehydrated, and then mounted with Malinol. The stained sections were evaluated by microscopy (DM‐2500; Leica Microsystems, Wetzlar, Hesse, Germany). We measured the SCG neuron body (long axis), number of nuclei, and shrunken neurons in 10 random areas of each section under a microscope (DM‐2500; Leica Microsystems) and modified the measurement methods of Liutkiene, Stropus, Pilmane, and Dabuzinskiene (2007).

### 2.3 Statistical analysis

The differences in the SCG measurement data were assessed, using two‐way analysis of variance (ANOVA) followed by the Bonferroni's post hoc test with one categorical independent variable and one continuous variable (the independent variable can consist of a number of groups). The level of significance was set as *p *<* *.05. The results are reported as the mean ± SD. The statistical analyses were performed, using the IBM SPSS Statistics Base, version 22 (Chicago, IL, USA).

## 3 Results

### 3.1 Macroscopic observations

**Figure 1:**
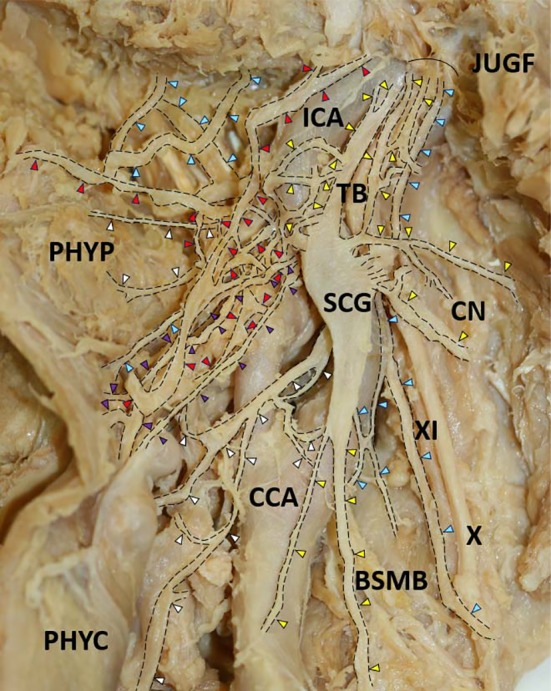
The complex SCG branch supply and communication with the cranial and cervical nerves. There are various branches, such as the branch of the internal carotid artery, glossopharyngeal nerve, inferior laryngeal nerve, pharyngeal nerve, branch of the pharyngeal constrictor, pharyngeal plexus of the vagus nerve, communicating branch of the carotid bifurcation, and internal and external carotid branches, observed in the lateral human neck region. Communication between each branch: *1, internal carotid branch; *2, communicating branch of the cervical nerve; *3, communicating branch of the vagus nerve; *4, communicating branch of the common carotid artery; *5, carotid branch; *6, communicating branch of the internal jugular vein. BSMB between the SCG and middle cervical ganglion; CN, cervical nerve; PHYC, pharyngeal constrictors; PHYP, pharyngeal plexus; TB tip branch; and theJUGF jugular foramen (left side, 94‐year‐old female)

**Figure 2:**
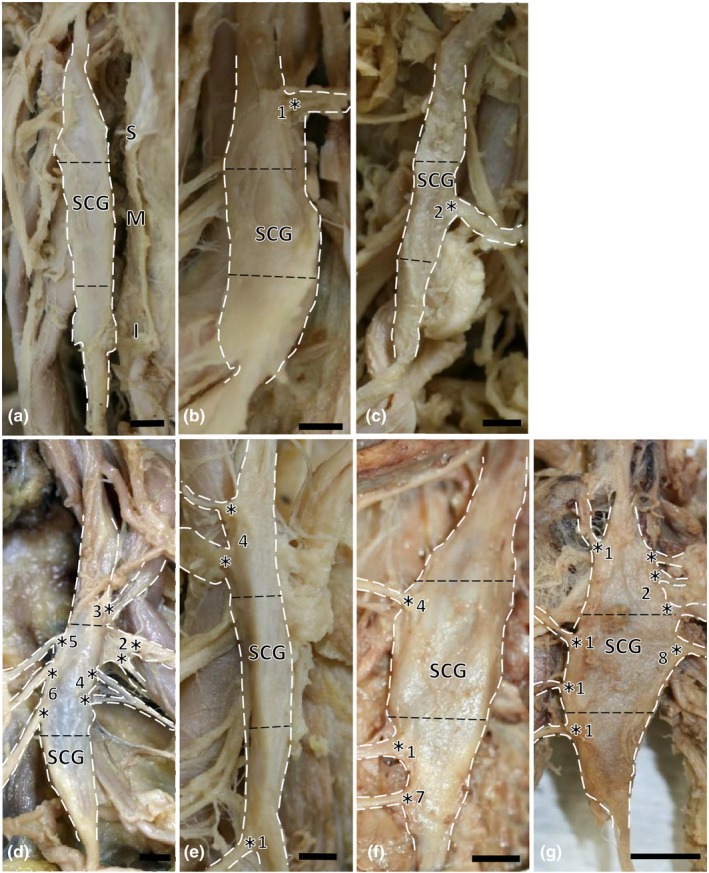
There are seven types of branches at the superior, middle, and inferior regions of the SCG. SCG, superior cervical ganglion. (a) Type I (no branch), left side, 77‐year‐old male; (b) Type II (superior region branch), right side, 78‐year‐old male; (c) Type III (middle region branch), left side, 84‐year‐old male; (d) Type IV (superior and middle region branches), left side, 87‐year‐old male; (e) Type V (superior and inferior region branches), right side, 79‐year‐old male; (f) Type VI (middle and inferior region branches), left side, 88‐year‐old female; (g) Type VII (superior, middle, and inferior region branches), right side, 88‐year‐old female. S = superior; M = middle; I = inferior region. Communication between each branch: *1, pharyngeal branch; *2, communicating branch of the cervical nerve; *3, communicating branch of the pharyngeal mucosa; *4, internal carotid branch; *5, communicating branch of the vagus nerve; *6, communicating branch of the superior laryngeal nerve; *7, laryngeal branch; *8, communicating branch of the internal jugular vein. Scale bar = 2 mm

The branch of the internal carotid artery and communication branch of the glossopharyngeal nerve were always found with finely complex arrangements at the top of the superior region in the SCG. The branch of the middle cervical ganglion and carotid branch from the inferior region of the SCG were also found in our examined specimens, and they descended to the basal portion of the neck and trunk. The carotid bifurcation and laryngeal branch mainly descended from the SCG, passed behind the internal and external carotid arteries, and supplied each organ. The complex carotid bifurcation branched from the SCG and also descended to the internal and external carotid arteries. They occasionally communicated with the rami of the glossopharyngeal nerve or directly supplied the carotid bifurcation and carotid branch. The branches of the inferior laryngeal nerve descended beneath the internal and external carotid arteries, ran into the larynx, and communicated with the inferior laryngeal nerve. At the anterior portion of the neck, the pharyngeal nerve was composed of some rami, which supplied the pharyngeal connective tissue and branch of the pharyngeal constrictor. They proceeded in a finely descending or ascending direction and communicated with the pharyngeal plexus of the vagus nerve, and then they supplied the superior and middle pharynxes. The internal carotid branch of the SCG also supplied the anterior portion of the neck. The communicating branch of the glossopharyngeal nerve of the SCG ran beneath the internal carotid artery after descending into the jugular foramen and communicating with the glossopharyngeal nerve. By contrast, some rami communicating branches of the vagus nerve and cervical nerve of the SCG ascended and communicated with the vagus nerve at the posterior region of the neck (see Figures 1 and 2).

We clearly identified the course of communication branches of the SCG to the vagus nerve, glossopharyngeal nerve, accessory nerve, internal carotid artery, internal jugular vein, external carotid artery, pharynx, pharyngeal mucosa, and larynx. The SCG communication branch consisted of the vagus nerve (14.5%, 93/641), communicating branch of the carotid bifurcation (3.4%, 22/641), branch of the pharynx (6.9%, 44/641), branch of the cervical nerve (C2‐C3) (6.9%, 44/641), communication branch of the superior laryngeal nerve of the vagus nerve (5.8%, 28/641), branch of the glossopharyngeal nerve (0.6%, 4/641), communicating branch of the jugular vein (0.9%, 6/641), and communicating branch of pharyngeal plexus on the pharyngeal mucosa (3.6%, 23/641). Moreover, the inferior glossopharyngeal nerve (11.2%, 72/641) and internal carotid branches (11.2%, 72/641) at the tip of the superior region of the SCG were always found in our examined specimens. Furthermore, the branch of the middle cervical ganglion (11.2%, 72/641) and cardiovascular branch (11.2%, 72/641) were also always found in the posterior region of the bottom SCG (Figure 2). We also compared the superior, middle, and inferior regions of the SCG; the SCG frequently supplied the inferior glossopharyngeal nerve (22.5%, 72/320) (expected frequency, 35.4), vagus nerve (18.1%, 58/320) (expected frequency, 45.8), and internal carotid branches (22.5%, 72/320) (expected frequency, 35.4) in the superior regions. The SCG also frequently supplied the superior laryngeal nerve of the vagus nerve (13.0%, 17/131) (expected frequency, 7.5), vagus nerve (19.1%, 25/131) (expected frequency, 18.7), cervical nerve (13.0%, 17/131) (expected frequency, 8.9), carotid branches (15.3%, 20/131) (expected frequency, 14.1), internal jugular vein (3.1%, 4/131) (expected frequency, 1.2), pharynx (15.3%, 20/131) (expected frequency, 8.9), and pharyngeal mucosa (6.9%, 9/131) (expected frequency, 4.6) in the middle region. The SCG frequently supplied the cardinal branch (37.9%, 72/190) (expected frequency, 22.0) and middle cervical ganglion (37.9%, 72/190) (expected frequency, 22.0) in the inferior region of the SCG. These communication nerves composed of 14 branches of the three regions of the SCG (χ^2^ = 598.47, df = 26, *p *<* *.001). Sex differences were only observed for the superior laryngeal nerve of the vagus nerve (male: 4.4%, 22/350; female: 2.1%, 6/291) (χ^2^ = 22.74, df = 13, *p *<* *.05). There was no difference between the right and left branches of the SCG (Table 1).

**Table 1:** SCG braches and communications at three region in donor Japanese cadavers

	I	II	III	IV	V
Superior	18.1% (58/320)	5.3% (17/320)	0.6% (2/320)	22.5% (72/320)	7.5% (24/320)
Middle	19.1% (25/131)	13.0% (17/131)	1.5% (2/131)	0.0% (0/131)	13.0% (17/131)
Inferior	5.0% (10/199)	1.5% (3/199)	0.0% (0/199)	0.0% (0/199)	1.5% (3/199)
Total	14.5% (93/641)	4.4% (28/641)	0.6% (4/641)	11.2% (72/641)	6.9% (44/641)
Male	14.5% (40/350)	4.4% (22/350)	0.6% (1/350)	11.2% (40/350)	6.9% (21/350)
Female	18.2% (53/291)	2.1% (6/291)	1.0% (3/291)	11.0% (32/291)	7.9% (23/291)

	VI	VII	VIII	IX	X	XI	XII	XIII	XIV
Superior	2.5% (8/320)	11.9% (38/320)	22.5% (72/320)	0.0% (0/320)	5.3% (17/320)	2.5% (8/320)	0.0% (0/320)	0.0% (0/320)	1.3% (4/320)
Middle	8.4% (11/131)	15.3% (20/131)	0.0% (0/131)	3.1% (4/131)	15.3% (20/131)	6.9% (9/131)	0.0% (0/131)	0.0% (0/131)	4.6% (6/131)
Inferior	1.5% (3/199)	6.0% (12/199)	0.0% (0/199)	1.0% (2/199)	3.5% (7/199)	3.0% (6/199)	36.2% (72/199)	36.2% (72/199)	4.5% (9/199)
Total	3.4% (22/641)	10.9% (70/641)	11.2% (72/641)	0.9% (6/641)	6.9% (44/641)	3.6% (23/641)	11.2% (72/641)	11.2% (72/641)	3.0% (19/641)
Male	3.4% (11/350)	10.9% (37/350)	11.2% (40/350)	0.9% (1/350)	6.9% (30/350)	3.6% (14/350)	11.2% (40/350)	11.2% (40/350)	3.0% (13/350)
Female	3.8% (11/291)	11.3% (33/291)	11.0% (32/291)	1.7% (5/291)	4.8% (14/291)	3.1% (9/291)	11.0% (32/291)	11.0% (32/291)	2.1% (6/291)

I, vagus nerve; II, superior laryngeal nerve; III, glossopharyngeal nerve; IV, inferior glossopharyngeal nerve; V, cervical nerve; VI, carotid bifurcation; VII, carotid artery; VIII, internal carotid artery; IX, vein X, pharynx; XI, pharyngeal mucosa; XII, cardiac branch; XIII, middle cervical ganglion; XIV, the others.

We also classified the branch pattern types from three portions—the superior region, middle region, and inferior region of the SCG: Type I (no branch) (1.4%, 1/72); Type II, superior region branch of the SCG (5.6%, 4/72); Type III, middle region branch of the SCG (6.9%, 5/72); Type IV, superior and middle regions branches of the SCG (37.5%, 27/72); Type V, superior and inferior region branches of the SCG (11.1%, 8/72); Type VI, middle and inferior region branches of the SCG (5.6%, 4/72); and Type VII, superior, middle and inferior region branches of the SCG (31.9%, 23/72). These types were obtained from the SCG branch, except for the internal carotid branch and communication branch of the glossopharyngeal nerve from the tip branch of the SCG, branch of the middle cervical ganglion and carotid branch between the SCG and middle cervical ganglion (Figure 2).

### 3.2 Measurement data and statistical analysis of the SCG

We obtained measurement data (length, width and thickness) of the SCG in the donated Japanese cadavers. Our three measured distances included JF‐SCG, JF‐HB, and JF‐CB, which are shown in Table 2. The measurement data of the SCG were almost the same as those in previous reports, except for T‐SCG (see Table 2). The measurement of T‐SCG was large compared with other previous data (Table 2). Moreover, a weak negative correlation between age and JF‐SCG or T‐SCG and L‐SCG was found in our results (*p *<* *.05). A moderate positive correlation between JF‐SCG and JF‐HB (*p *<* *.01) or JF‐CB (*p *<* *.01), as well as between JF‐CB and JH‐ HB (*p *<* *.01) was detected. Sex differences were also observed for L‐SCG (*p *<* *.01), JF‐SCG (*p *<* *.001), JH‐HB (*p *<* *.01) and JF‐CB (*p *<* *.01). Statistical analysis indicated no correlation data for the communication branches of the SCG to the vagus, glossopharyngeal, or accessory nerves, internal carotid artery, internal jugular vein, external carotid artery, pharynx, or larynx.

**Table 2:** Measurement data of SCG in donor Japanese cadavers (mm)

	L‐SCG	W‐SCG	T‐SCG	JF‐SCG	JF‐HB	JF‐CB
Male, Right side	27.62 ± 3.88	8.05 ± 1.52	3.67 ± 1.16	34.1 ± 5.32	68.82 ± 9.3^b^	56.59 ± 9.69
Male, Left side	28.26 ± 4.24	7.83 ± 1.74^a^	3.51 ± 0.86	34.93 ± 7.54	69.28 ± 6.82^b^	59.88 ± 5.46
Female, Right side	26.87 ± 2.86^b^	8.55 ± 1.4	3.72 ± 0.86	28.33 ± 6.7	64.19 ± 12.09	52.24 ± 10.94
Female, Left side	22.88 ± 5.32^b^	8.41 ± 1.76^a^	4.02 ± 0.84	26.38 ± 6.5	59.31 ± 6.95	50.54 ± 8.35

SCG, superior cervical ganglion; L, length; W, width; T, thickness; JF, jugular foramen; HB, hyoid bone; CB, carotid bifurcation.

All differential expressions are statistically significant at *p *<* *.01.

a No differential expressions.

b Differential expressions are statistically significant at *p *<* *.05.

### 3.3 Histochemical observations

**Figure 3:**
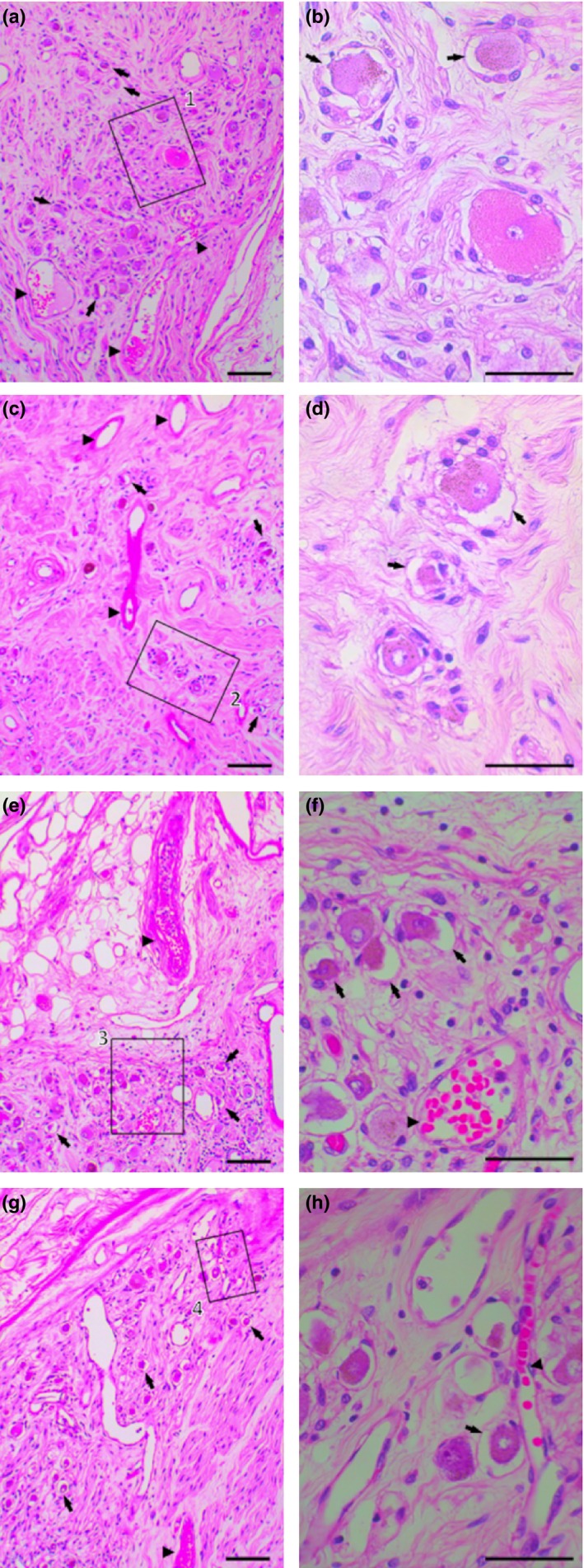
Histological characteristics of the superior, middle, and inferior regions of the human SCG as revealed by hematoxylin and eosin staining. (a) Elongated myelin bundle connected and inserted into the SCG with numerous ganglion cells located at the superior region of the SCG. (b) Ganglion cells and shrunken neurons were also found in the large magnification of square 1 (a). (c) A large number of elongated and oval capillary vessels were found around the ganglion cells in the middle region of the SCG. (d) An assembly of shrunken neurons formed cell masses that were found in the middle region of the SCG in the large magnification of square 2 (c). (e) Elongated myelin bundle inserted into the middle region of the SCG in which numerous ganglion cells were located. (f) Large oval capillary vessels were located around ganglion cell masses in the large magnification of the square (e). (e) Numerous ganglion cells located at the inferior region of the SCG. (h) Shrunken neurons were found near the capillary vessels in the large magnification of square 3 (g). Shrunken neurons (black arrow), blood vessel (black arrowhead), Scale bar = 1 μm (a, c, e, g); Scale bar = 0.5 μm (b, d, f, h) (left side, 78‐year‐old male)

In the sagittal sections of the SCG, the rami fiber bundle of the inferior branch was inserted into the lateral side of the SCG, and it was composed of compressed meandering fibers (Figure 3). We mainly observed the myelin bundle of the insertion region, which connected with other nerves at three regions (superior, middle and inferior regions) (see Figure 3). The assembly of neurons formed a cell mass in three regions of the SCG (Figure 3a–h). The neurons were also surrounded by numerous round‐like myelin bundles, which formed a cell circle (Figure 3). In the superior region, ganglion cells were found near the meandering myelin fiber bundle (Figure 3a,b). The neurons were also surrounded by numerous round‐like myelin bundles, which formed a cell circle that was apparent under high magnification in Figure 3a (Figure 3b). A large number of shrunken neurons were also found that were scattered around the myelin bundle area of the superior region of the SCG (Figure 3a,b). In the middle region of the SCG, a large number of elongated and oval capillary vessels were found around the ganglion cell mass and myelin bundle (Figure 3c,d). Large and oval capillary vessels in the inner side of the middle region were also found compared with those in the superior region (Figure 3c,b). The assembly of neurons formed cell masses that were mostly found in the middle region of the SCG compared with other regions (Figure 3a–h). The large elongated capillary vessels were located around large ganglion cell masses in the inferior region of the SCG (Figure 3c,d). In particular, numerous shrunken neurons were also found in the ganglion cell masses (Figure 3c–f). These morphological features were also present in the lateral side of the middle region and inferior region of the SCG (Figure 3g,h).

### 3.4 Statistical analysis of the ganglion cells

The number of ganglion cells with large neurons per unit area (1 mm^2^) in the SCG was the highest in the middle region (476 ± 49.5, 37.2%) of the SCG, followed by the superior region (471 ± 38.5, 36.8%), and finally the inferior region (333 ± 36.0, 26.0%). The number of shrunken neurons per unit area (1 mm^2^) in the SCG was the highest in the middle region (233 ± 40.2, 48.9%) of the SCG, followed by the superior region (169 ± 32.0, 35.8%), and finally the inferior region (116 ± 25.4, 34.8%).

## 4 Discussion

### 4.1 SCG supplements

In the anatomical textbooks, the petrous ganglion of the glossopharyngeal nerve communicates with the superior cervical ganglion (SCG) of the auricular branch and jugular ganglion of the vagus and facial nerves (Robinson, 1922). Theses nerve distributions are complex due to both the domination of the cranial nerve and spinal nerve and the sympathetic and parasympathetic nerve supply at these sites. Moreover, there is particularly complex communication between these cranial nerves. Therefore, we needed to define each communication between the SCG and cranial and cervical nerves. The GPN communicates with the SCG, vagus nerve, cervical nerve (C1‐C2), and lingual nerve (LN). The GPN is joined by branches of the SCG to form the loop between the first and second cervical nerves (Standring, 2005). Medial branch rami are composed of the cervical ganglion and laryngopharyngeal and carotid branches. Communicating rami connect to the cardiac plexus, and the pharyngeal plexus also connects to the glossopharyngeal and vagal rami. The anterior branch communicates with the common and external carotid arteries and supplies the submandibular ganglion (Standring, 2005). In our observations, branches of three portions of the SCG connected to the glossopharyngeal nerve, cervical nerve, and vagal nerve. We defined those branches that differed at three regions: the superior, middle, and inferior regions of the SCG. Many of the branches from the middle of the SCG were also found to be distributed in cervical nerves and the vagus nerve. Moreover, the JF‐SCG was shorter in samples from aged subjects, and sex differences were found in JF‐CB, JF‐HB, JF‐SCG and L‐SCG in our analysis. These results provided useful information for nerve block. The branches emerging from the SCG are also important targets for anesthetic treatment (Elias, 2000; Siegenthaler et al., 2013; Wirz et al., 2008). Nerve block treatments have demonstrated that these branches also affect the final distribution areas of the glossopharyngeal nerve, cervical nerves and vagus nerve, suggesting that the fibers connect to the carotid bifurcation and aortic bodies.

### 4.2 Communication between the SCG branch and u*p*per vertebrae region (C1‐C3)

In general, cervical sympathetic fibers pass through the stellate ganglion and run to the vertebral and internal carotid arteries from the SCG and stellate ganglion (Mitchell et al., 2009). In clinical treatment, the application of ganglionic local opioid analgesia (GLOA) at the SCG can represent a suitable and simple treatment option for neuropathic facial pain (Elsner et al., 2006). Moreover, the localization of the common carotid artery with the SCG is important in the anesthetic block procedure (Jonathan, Stark, Safir, & Rahman, 2012). The SCG block is needed for anesthetic block in clinical treatment. In our results, the SCG was located at C2. Previous reports have indicated that the SCG was also located between C2 and C3 (see Table). Moreover, a morphological structure is an important element for a recognized location of the SCG. The GPN communicates with the SCG, vagus nerve, cervical nerve (C1‐C2), and lingual nerve (LN), which were also found at the site of the neck. In the atlas, the GPN was joined by branches of the SCG from the loop between the first and second cervical nerves (Standring, 2005). Therefore, there were various connections with cervical spinal nerves from C1 to C2. Many previous studies described joint communication sites between the SCG and cervical nerves (References). Previous reports indicated that the SCG branch communicated with the cervical nerve C2 branch (Kiray, Arman, Naderi, Güvencer, & Korman, 2005; Saylam, Ozgiray, Orhan, Cagli, & Zileli, 2009; Fazliogullari et al., 2016). By contrast, the communication branch links the cervical nerve C1 and C2 branches (Hara, Tanuma, & Suzuki, 1993; Matz, Pritchard, & Hadley, 2007; Robinson, 1922; Spacek, Hanl, Groiss, Koinig, & Kress, 1998). The branch was also found to communicate with the cervical nerve C2 and C3 branches (Leonhardt, Töndury & Zilles^1987^; Elias, 2000) or C3 branch (Siegenthaler et al., 2013). Moreover, the communication branch was complex; the SCG‐communicated cervical nerve C3 branch is 44% (11/25 samples) (Pick & Sheehan, 1946) and 12.3% (130/1,054 samples) (Hoffman, 1957), and the C4 branch is 12% (3/25 samples) (Pick & Sheehan, 1946). The branch communication suggested that there may be a considerably long distance from the cervical nerve C1 to C4. This communication was found from cervical nerves C2‐C3 (72 samples) in our observations. We tried to examine the joint site after dissection at the C1 to C4 connection. Therefore, we defined the joint site from C1 to C3 and then identified that the communicated joint was mainly located at C2. The communication branch between the SCG and cervical nerve was fairly thick. In general, the cervical nerve formed a plexus with rami of the four cervical nerves (C1 to C4 cervical segments) in the neck (Standring, 2005). It was composed of a coalescence of four ganglia linked to the upper four cervical nerves, C1‐C4. These preganglionic neurons then entered the SCG and synapse with the postganglionic neurons that leave the rostral end of the SCG and innervate target organs of the head and neck (Standring, 2005). That is, cervical nerves contain many sympathetic nerves after leaving the SCG that connect to the head and neck muscles, carotid bifurcation, sympathetic ganglion of the salivary gland, common carotid artery, internal jugular vein and connective tissue. The efficacy of ganglionic local opioid analgesia (GLOA) at the superior cervical ganglion (SCG) can represent a suitable and simple treatment option for neuropathic facial pain (Elsner et al., 2006). Moreover, our analysis indicated that in a large number of cases, the superior region of the SCG branch is connected to the cervical nerve. This site had a low number of shrunken neurons, suggesting that this site is not affected by aging compared with the middle region, which had a large number of ganglion cells. Therefore, the location and structure of the jointed site of the cervical nerve is important for safe and efficient anesthetic block in clinical treatment.

### 4.3 Communication among SCG branches, the glossopharyngeal nerve and the vagus nerve

In general, the communication between the glossopharyngeal nerve and vagus nerve was found at the dorsolateral surface of the pharynx; this nerve connects to the inferior pharyngeal constrictor muscle, palatopharyngeus muscle, salpingopharyngeus muscle, middle pharyngeal constrictor muscle and inferior pharyngeal constrictor muscle (Sakamoto, 2009). The pharyngeal plexus was formed from the pharyngeal branch of the glossopharyngeal nerve and vagus nerve and contained SCG fibers (Standring, 2005). Our results suggest that SCG fibers connect to the glossopharyngeal nerve at the upper region of the SCG and to the vagus nerve at the middle region of the SCG (see Figure 1). The different compositions of the SCG nerve varied between the vagus nerve and glossopharyngeal nerve; a large number of branches of the three regions of the SCG (14.5%, 93/641) were found in the connection of the vagus nerve, and a moderate number of branches of the SCG were found in the connection of the glossopharyngeal nerve to the SCG (11.2%, 72/641) in our results (see Table 1). These fibers were related to the formation of the pharyngeal plexus. The communication site of these nerves is an important landmark in the region of the head and neck during surgery. In head and neck surgery, the locations of the blood vessels (Hayashi et al., 2005; Jeganath, McElwaine, & Stewart, 2001), lymph nodes (Jones, Roland, Field, & Phillips, 1994; Sugenoya et al., 1993) and recurrent laryngeal nerve (Balanzoni, Altini, Pasi, & Fussi, 1994; Picucci et al., 1997) are also important. The communication between the vagus nerve and SCG may affect the blood flow of the carotid artery; damage to the recurrent laryngeal nerve may lead to hoarseness. Our data indicate that several vagus communication branches are located in the superior region of the SCG. Some branches are ancillary, and the measurement data of the SCG communication branch may affect head and neck surgery. Moreover, there are many SCG branches that connect to the human heart (Ellison & Williams, 1969; Hara et al., 1993; Lemere, 1932). The frequency of the occurrence of this carotid branch is high (81.3%: Fukuyama, 1982; 67; %: Ellison & Williams, 1969; 100; %: Hara et al., 1993). There are also multiple variations of this branch, which communicates with the middle ganglion and superior laryngeal nerves that descend to the heart. In our results, the carotid branch was mainly located in the inferior SCG region; however, many communication branches of the superior laryngeal nerve and common carotid artery may connect to the heart. However, we observed a complex supply to the carotid artery at the SCG in our previous report (Sato et al., 1997). There are three branches that connect to the heart: the carotid artery, carotid bifurcation and SLN branches. In particular, the inferior glossopharyngeal nerve (11.2%, 72/641) and internal carotid artery branch (11.2%, 72/641) are located in the superior region in our results (see Table 1). Therefore, the communication branches of the glossopharyngeal nerve mainly connect to the carotid artery. The location of the communication site between the glossopharyngeal nerve and SCG indicates the importance of the right‐sided lateralization of the head and neck compared with previous reports (Saylam et al., 2009).

### 4.4 Distribution at the superior, middle, and inferior regions of the SCG

Previous reports have indicated that the size and number of neuronal somata are related to the size of an organ (Wirz et al., 2008). By contrast, the size of neuronal somata does not correlate with the size of target organs in the rat superior cervical ganglion (SCG) and stellate ganglion (STG) (Asamoto, Tamamaki, & Nojyo, 1997). In our results, numerous shrunken neurons were found, mainly near the myelin bundle in the superior and inferior regions of the SCG. Liutkiene et al. (2007) suggested a decreased number of myelin fibers and larger neurons in aged human subjects. Small neurons, a decreased number of neurons and increased lipopigment were apparent in the SCG of aged mice compared with young mice (Lahtivirta, Koistinaho, & Hervonen, 1992). By contrast, Schmidt (2002) suggested that SCG neurons in rodents do not exhibit changes in neuro‐axonal dystrophy as a function of age compared with prevertebral superior mesenteric ganglions. In our results, the morphological features of the human neuron exhibited different changes in three locations of the SCG. In particular, ganglion neurons of the superior and inferior regions exhibited changes in features with shrunken neurons and capillary vessels compared with the middle region of the SCG. These results suggested that branches of the vagus nerve, communicating branch of the carotid bifurcation, branches of the pharynx, communication branches of the cervical nerve, communication branches of the superior laryngeal nerve, internal and external carotid branches of the SCG, and branches of the glossopharyngeal nerve were affected by aging. Our histological analysis revealed that a large number of ganglion cells in the middle region of the SCG had a large number of shrunken neurons compared with other regions. The middle region of the SCG was affected by aging, suggesting some inferences to the communicating branch of the carotid bifurcation, internal carotid branch and pharyngeal plexus of the vagus nerve. The SCG exerts effects on vagus nerve function in the neural system. Moreover, a few pharyngeal nerves (3.1%. 20/641), carotid branches (3.1%, 20/641) and cervical nerves (2.7%, 17/641) were found in the middle region of the SCG (modified from Table 1). Therefore, these sites indicate an effect of aging. In our analysis of the number of SCG branches, there are differences in the three regions of the SCG, especially in the connections to the glossopharyngeal nerve, vagus nerve, and internal carotid branches of the SCG in the superior regions compared with other regions (χ^2^ = 587.72, df = 26, *p *<* *.001).

## 5 Conclusion

Cervical nerves contain many sympathetic nerves after leaving the SCG that connect to head and neck muscles, the carotid bifurcation, the sympathetic ganglion of the salivary gland, the common carotid artery, the internal vein and connective tissue. The communication site mainly of the cervical nerve, vagus nerve and glossopharyngeal nerve is an important landmark in head and neck anatomy. There are differences in the three regions of the shrunken neurons of the SCG. The communication between the vagus nerve and SCG may affect the blood flow of the carotid artery and hoarseness related to the recurrent laryngeal nerve. Finally, the communication branches that mainly connect to the vagus nerve and glossopharyngeal nerve provide useful data for clinical treatments, head and neck ganglion block, surgical treatments, or guides for nerve systems.

## Conflicts of interest

The authors declare that there are no conflicts of interest.
